# Therapeutic Potential of *Irpex lacteus* Polysaccharides in Lupus Nephritis: Insights From Gut Microbiota and Metabolomics Analysis in MRL/Lpr Mice

**DOI:** 10.1002/fsn3.71446

**Published:** 2026-01-09

**Authors:** Guoxin Ji, Cuicui Li, Zhuangzhuang Yao, Zhimeng Li, Bo Yang, Liming Hu, Hang Yu, Tongwei Jiang, Shumin Wang, Huan Wang

**Affiliations:** ^1^ College of Pharmacy Changchun University of Chinese Medicine Changchun China; ^2^ College of Integrated Chinese and Western Medicine Changchun University of Chinese Medicine Changchun China; ^3^ State Key Laboratory of Natural and Biomimetic Drugs, School of Pharmaceutical Sciences Peking University Beijing China; ^4^ College of Traditional Chinese Medicine Changchun University of Chinese Medicine Changchun China; ^5^ Jilin Ginseng Academy Changchun University of Chinese Medicine Changchun China

**Keywords:** gut microbiota, *Irpex lacteus* polysaccharides, lupus nephritis, metabolites, systemic lupus erythematosus

## Abstract

Polysaccharides from *Irpex lacteus* (PCP) were evaluated for their therapeutic effects on lupus nephritis (LN) in MRL/lpr mice. After 8‐week interventions with low‐ and high‐dose PCP, we systematically evaluated the therapeutic efficacy by measuring the levels of autoantibodies, the expression of inflammatory cytokines, and renal function‐related parameters. Finally, 16S rDNA gut microbiome sequencing with metabolomics analysis was used to explore the pharmacological mechanism of PCP intervention in LN. PCP could reverse the phenotype of MRL/lpr mice, reduce autoantibody levels, alleviate inflammatory responses, and improve renal function. Gut microbiome analysis found that PCP can improve gut microbiota composition and abundance of two phyla (*Firmicutes*, *Bacteroidota*) and five genera (*Lachnospiraceae* NK4A136 group, *Alistipes*, *Butyricicoccus*, *Bacteroides*, *Lactobacillus*), which play an important role in the process of PCP intervention on metabolism in MRL/lpr mice. UHPLC–MS untargeted metabolomics showed that PCP significantly affects multiple key differential metabolites, including Linoleic acid, L‐Phenylalanine, L‐Tyrosine, and 56 other metabolites. These metabolites are primarily involved in metabolic pathways such as tryptophan metabolism, phenylalanine, tyrosine and tryptophan biosynthesis, tyrosine metabolism, linoleic acid metabolism, and arachidonic acid metabolism. Correlation analysis between gut microbiota and differential metabolites reveals a close relationship, suggesting that gut microbiota promoting host metabolism may be one of the mechanisms by which PCP treats LN. PCP alleviates LN by modulating the “microbiota‐metabolism axis,” reducing autoantibodies, inflammation, and renal damage, while reshaping gut microbiota and regulating key metabolic pathways.

## Graphical abstract

Polysaccharides from *Irpex lacteus* alleviated kidney inflammatory response. PCP could reverse the phenotype of MRL/lpr mice, reduce autoantibody levels, alleviate inflammatory responses, and improve renal function. Improving the body's metabolism by gut microbes may be the possible mechanism of PCP in treating LN.

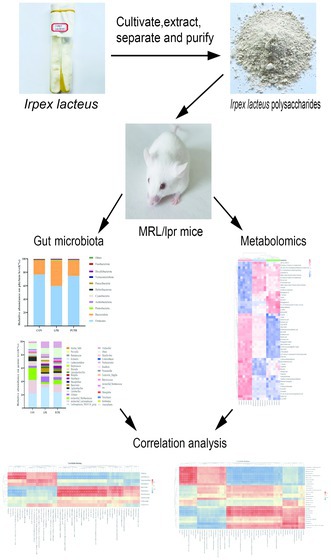

## 1 Introduction

Systemic Lupus Erythematosus (SLE) is a chronic autoimmune disease characterized by multi‐system involvement. Its core pathological mechanism involves the abnormal activation of autoreactive B cells and the overproduction of pathogenic autoantibodies, such as antinuclear antibodies (ANA) and anti‐double‐stranded DNA antibodies (anti‐dsDNA) (Alforaih et al. 2022). These autoantibodies form circulating immune complexes (CIC) that deposit in target organs, including the skin, joints, and kidneys, activating the complement system and recruiting inflammatory cells such as neutrophils and macrophages, ultimately leading to multi‐organ damage (Hanly et al. 2016). Notably, over 50% of SLE patients develop lupus nephritis (LN), one of the most severe complications of SLE and a leading cause of end‐stage renal disease and patient mortality (Anders et al. 2020; Reppe Moe et al. 2019). In terms of clinical treatment, the clinical treatment of LN is based mostly on therapies such as immunosuppressants or hormones to suppress the immune responses to “self‐antigen,” which cannot completely alleviate the disease progression (Mohamed et al. 2019). Moreover, long‐term and high‐dose use can cause many toxic side effects, leading to a serious impact on the survival and quality of life of patients (Guo et al. 2020). Therefore, the development of novel and efficacious therapeutic regimens that modulate immune function and control inflammatory responses is of paramount importance for the treatment and improvement of the condition and prognosis of LN.

The gut microbiome, often regarded as a newly recognized metabolic organ, plays a crucial role in regulating host metabolism. Their composition and abundance can be varied, depending on internal factors (e.g., host genetics) and external factors (e.g., diet, lifestyle, and drugs) (Sommer and Bäckhed 2013). The change of gut microbiome composition has been reported to modulate the progression of many human diseases, such as non‐alcoholic fatty liver disease, type 2 diabetes, and cancer (Canfora et al. 2019). Accumulating evidence has proved that metabolites reside at the important interface between gut microbiome and the host health status (Levy et al. 2016; Tong et al. 2020). In LN, changes in gut microbiota have been documented, highlighting the potential link between these microbial communities and the disease's pathogenesis (de la Visitación et al. 2021; Luo et al. 2018). This has positioned gut microbiota as a significant area of interest in LN research. However, the interplay between the gut microbiome and metabolic processes in LN patients remains to be fully elucidated. Conducting an integrated analysis of both the gut microbiome and metabolome could provide valuable insights into the intricate nature of LN.

Microbiota‐targeted therapy has become a frontier in autoimmune disease research. The gut microbiota significantly influences drug efficacy by regulating host immunity, metabolism, and barrier functions. Microbiota interventions can enhance the therapeutic effects of immunosuppressants and improve disease activity through the metabolite‐immune axis (Huang et al. 2023). In the field of traditional Chinese medicine (TCM), plant polysaccharides often exert holistic regulatory effects through the “gut‐kidney axis.” On one hand, plant polysaccharides act as prebiotics to selectively promote the proliferation of beneficial bacteria; on the other hand, their microbial metabolites can enhance the gut barrier, forming a “polysaccharide‐microbiota‐host” bidirectional positive feedback regulation (Xia et al. 2022; Yin et al. 2022). This multi‐target regulatory network provides a new perspective for the treatment of LN. Furthermore, elucidating its molecular mechanisms in conjunction with metabolomics will significantly advance modern research based on the microbiota.

*Irpex lacteus*, a white‐rot basidiomycete, is widely used in environmental remediation for its robust lignin‐degrading capabilities, particularly in the degradation of recalcitrant organic pollutants in contaminated soils and the decolorization of synthetic dyes. In recent years, its medicinal and edible value has garnered increasing attention, especially its key bioactive component—*Irpex lacteus* polysaccharides (PCP), which have been demonstrated to possess multiple biological activities, including anti‐fatigue, anti‐inflammatory, and immunomodulatory effects. Research indicates that PCP inhibits the proliferation of mesangial cells, providing a new clinical basis for the prevention and treatment of mesangial proliferative diseases and glomerulonephritis (Wang et al. 2016). Furthermore, PCP has shown significant therapeutic effects in models of membranous nephropathy (Han et al. 2020). Notably, beyond its known efficacy in chronic glomerulonephritis, PCP also exhibits remarkable therapeutic potential in the treatment of LN, opening new avenues for its application in kidney diseases.

This study aims to systematically evaluate the therapeutic effects of PCP on the MRL/lpr mouse model, focusing on its impact on cytokine levels and renal pathological changes. Furthermore, the research will explore the regulatory effects of PCP on gut microbiota and metabolic profiles to elucidate its underlying mechanisms in the treatment of LN. The findings are anticipated to offer new strategies for LN treatment and promote the application of PCP in the development of functional foods and pharmaceuticals.

## 2 Materials and Methods

### 2.1 Preparation of PCP

The strain of *Irpex lacteus* used in this experiment was sourced from the China Committee for Culture Collection of Microorganisms, and the specific strain was selected from the preserved collection. The experiment began with the activation of *Irpex lacteus* cultured on a slant, followed by its transfer to a liquid medium for expansion. Through multiple subcultures, a genetically stable strain was obtained. This stable strain was then inoculated into a fermenter for fermentation. After fermentation, the culture was subjected to three rounds of extraction using 10 times the volume of water to fully extract the target components. The extract was treated with ethanol precipitation, and protein impurities were removed using the Sevag method. Ultimately, the purified extract was dialyzed and lyophilized to yield high‐purity Irpex lacteus polysaccharides (PCP).

### 2.2 Physical and Chemical Analyses of PCP

The total PCP content was evaluated using the well‐established phenol‐sulfuric acid method, with glucose serving as the standard for comparison. The soluble glycoprotein content was quantified through Coomassie brilliant blue staining. Sulfate content was measured using barium chloride gelatin turbidimetry, employing bovine serum albumin and the sulfate radical as respective standards.

The purity of PCP was assessed by determining its total polysaccharide content, expressed as a percentage of the total sample mass. Furthermore, to ensure batch‐to‐batch consistency, the main components of PCP were characterized using Ultraviolet (UV), Fourier‐transform infrared (FT‐IR) spectroscopy, and High‐Performance Anion‐Exchange Chromatography (HPAEC).

For ultraviolet (UV) absorption spectrometry, a 0.5 mg/mL solution of PCP in deionized water was scanned over the wavelength range of 190–400 nm using a UV–Vis spectrophotometer (manufactured by Shimadzu Experimental Equipment Co. Ltd., Shanghai, China) with a 1 nm scan interval. Infrared (IR) absorption spectroscopy was conducted using a Nicolet iZ‐10 spectrometer (Thermo Nicolet, USA). PCP samples were mixed with KBr powder and compressed into 1 mm pellets for Fourier‐transform infrared (FT‐IR) measurements spanning from 4000 to 400 cm^−1^.

Monosaccharide compositions were determined using high‐performance anion‐exchange chromatography (HPAEC) with a CarboPac PA‐20 anion‐exchange column (3 × 150 mm; Dionex) and a pulsed amperometric detector (PAD; Dionex ICS 5000+ system). The chromatographic conditions were as follows: flow rate, 0.5 mL/min; injection volume, 5 μL. The solvent system consisted of solvent A (ddH2O), solvent B (0.1 M NaOH), and solvent C (0.1 M NaOH, 0.2 M NaAc). The gradient program was set as follows: the volume ratio of solutions A, B, and C was 95:5:0 at 0 min, 85:5:10 at 26 min, 85:5:10 at 42 min, 60:0:40 at 42.1 min, 60:40:0 at 52 min, 95:5:0 at 52.1 min, and 95:5:0 at 60 min.

### 2.3 Animals and Experimental Design

Eight‐week‐old BALB/c and MRL/lpr mice were sourced from SPF Biotechnology Co. Ltd. (Beijing, China), holding the license number SCXK (Jing) 2019‐0010. All experimental protocols were conducted in strict accordance with the guidelines set by the Ethics Committee of Changchun University of Chinese Medicine, under the ethical approval number 2023515. The mice were maintained in a barrier facility, under standard housing and dietary conditions. Following a one‐week acclimatization period, the BALB/c mice were designated as the control group (CON). The MRL/lpr mice were divided randomly into four groups of six mice each: a model group (LPR), a positive control group (PAT, receiving 5 mg Prednisone acetate tablet/kg body weight), a low‐dose PCP group (PCPL, receiving 1 g PCP/kg body weight), and a high‐dose PCP group (PCPH, receiving 2 g PCP/kg body weight). The dose of PCP was determined based on the effective dose range for kidney disease reported in previous studies (Dong et al. 2017; Li et al. 2018). The treatment duration of 8 weeks was selected to cover the critical period of autoantibody elevation and kidney injury in MRL/lpr mice, ensuring an adequate evaluation of the intervention's effects (Liu et al. 2022; Wang, Zhu, et al. 2021). The PCP and PAT groups received their respective treatments via gavage, while the control and model groups received an equivalent volume of distilled water by gavage. The experiment spanned 8 weeks, with urine samples collected biweekly for protein analysis, resulting in five collection points. At the conclusion of the 8‐week study, the mice were humanely euthanized. Blood serum was obtained by centrifugation at 3000 rpm for 10 min. The abdominal cavity was opened aseptically to access the internal organs. A segment of the ileum was excised, and a 1 cm portion was used to collect fecal contents into a sterile tube. All samples were promptly stored at −80°C, with rigorous aseptic techniques observed throughout the process.

### 2.4 Enzyme‐Linked Immunosorbent Assays (ELISA)

Urine protein (UP), anti‐double‐stranded DNA antibody (anti‐dsDNA), anti‐nuclear antibody (ANA), anti‐Smith antibody (anti‐Sm), interleukin‐10 (IL‐10), tumor necrosis factor‐alpha (TNF‐α), interleukin‐6 (IL‐6), interleukin‐17 (IL‐17), blood urea nitrogen (BUN), creatinine (Cr), complement component 3 (C3), and complement component 4 (C4) levels were measured following the manufacturer's instructions provided with the kits (Nanjing Jiancheng Group, China).

### 2.5 Histopathological Analysis of Kidney Tissue

Kidney tissue, initially fixed in 4% paraformaldehyde, underwent a series of dehydration steps using an alcohol gradient, followed by immersion in paraffin wax. The wax‐embedded tissue was subsequently frozen and trimmed, and the cooled wax blocks were placed in a paraffin microtome. Sections with a thickness of 4 μm were carefully mounted onto glass slides. Hematoxylin and eosin (H&E) staining and Masson's trichrome staining were then applied to these sections. Following staining, the sections were scanned, allowing for detailed observation of the kidney tissue pathology.

### 2.6 IgG Immunofluorescence Staining

The dewaxing and dehydration steps were carried out using xylene and a series of graded alcohols. Antigen retrieval was achieved using an EDTA‐based antigen retrieval buffer. Following a 30‐min blocking step with BSA, the primary antibody was applied and allowed to incubate overnight at 4°C. Subsequently, the slides were washed three times for 5 min each with PBS. The corresponding secondary antibody was then applied and incubated at room temperature for 1 h. The cell nuclei were counterstained with DAPI, and the sections were treated with an anti‐fluorescence quenching agent. Finally, the slides were scanned or photographed for detailed observation.

### 2.7 16S rRNA Gene Sequencing

Total genomic DNA was extracted using the TGuide S96 magnetic bead‐based soil/feces DNA extraction kit (Tiangen Biotech, Beijing) according to the manufacturer's instructions, with DNA integrity verified by 1.8% agarose gel electrophoresis and concentration measured using a NanoDrop 2000 spectrophotometer (requiring OD260/280 ratios between 1.8 and 2.0). For high‐resolution taxonomic classification, full‐length 16S rRNA gene amplification was performed using universal primers 27F (5′‐AGRGTTTGATYNTGGCTCAG‐3′) and 1492R (5′‐TASGGHTACCTTGTTASGACTT‐3′) with sample‐specific 10‐bp PacBio barcodes incorporated at the 5′ end for multiplex sequencing demultiplexing. PCR amplification was conducted using KOD One PCR Master Mix (TOYOBO) in 25 μL reaction volumes under the following conditions: initial denaturation at 95°C for 2 min; 25 cycles of denaturation at 98°C for 10 s, annealing at 55°C for 30 s, and extension at 72°C for 90 s; followed by final extension at 72°C for 2 min, with negative controls included in each batch to monitor contamination. The purified PCR products were processed using VAHTS DNA Clean Beads (Vazyme) for purification, and libraries were constructed with the SMRTbell Express Template Prep Kit 2.0, followed by sequencing on the PacBio Sequel II platform using Sequel II Binding Kit 2.0, achieving an average depth of 50,000 reads per sample with raw data filtered (quality score > Q20) through SMRT Link software. Bioinformatic analysis on the BMKCloud platform included primer removal using Cutadapt, OTU clustering at a 97% similarity threshold with the USEARCH algorithm, taxonomic annotation against the SILVA database (v138), and removal of singleton OTUs and chimeric sequences, with alpha diversity (Chao1, Shannon) and beta diversity (PCoA) analyses performed as described in Section 2.9.

### 2.8 Serum Untargeted Metabolomics

The LC–MS system for metabolomics combines a Waters Acquity I‐Class PLUS UPLC with a Xevo G2‐XS Q‐TOF mass spectrometer. Chromatographic separation uses a Waters Acquity UPLC HSS T3 column with a gradient elution protocol. The Q‐TOF operates in MSE mode, acquiring primary and secondary MS data at low and high collision energies, with a scanning frequency of 0.2 s/spectrum. ESI parameters include specific capillary and cone voltages, ion source and desolvation temperatures, and nitrogen gas flows. Raw data from MassLynx V4.2 is preprocessed via Progenesis QI for peak alignment, normalization, and annotation against METLIN and custom libraries. Quality control involves PCA, Spearman correlation, and OPLS‐DA modeling with permutation tests. Differential metabolites are screened using specific thresholds, and pathway enrichment analysis is conducted using hypergeometric testing on KEGG, HMDB, and Lipid Maps databases. This workflow ensures high‐confidence metabolite identification and quantitation with rigorous statistical validation.

### 2.9 Statistical Analysis

Data are presented as mean ± SD from independent replicates. Statistical analyses were performed using GraphPad Prism 9.0 and R 4.0.3. For group comparisons, one‐way ANOVA (with Tukey's post hoc test) or Kruskal‐Wallis test (with Dunn's post hoc test) was applied based on data distribution. Microbiome alpha diversity was analyzed by Kruskal‐Wallis test, while beta diversity was assessed by PERMANOVA based on Bray‐Curtis distance. Differential species and metabolites were identified using LEfSe (LDA > 4, *p* < 0.05) and OPLS‐DA (VIP > 1, FC > 1.0, *p* < 0.05), respectively. Correlations were calculated by Spearman's rank test (|ρ| > 0.5). A P‐value of less than 0.05 was considered statistically significant, while a P‐value of less than 0.01 was considered highly statistically significant.

## 3 Results

### 3.1 PCP Physicochemical Characteristics

Table 1 summarizes the contents of polysaccharide, soluble glycoprotein, and sulfate within PCP. The polysaccharide content was the highest at 696.86 ± 6.54 mg/g, followed by sulfate radical at 94.40 ± 1.36 mg/g and soluble protein at 11.51 ± 0.08 mg/g. This indicates a purity level of 69.69% for the PCP.

**TABLE 1:** Chemical components of PCP.

Physicochemical indexes	Standard curve	Correlation coefficient	Content (mg/g)
Polysaccharide	*Y* = 379.44x − 26.778	0.9995	696.86 ± 6.54
Soluble protein	*Y* = 1.1688x + 0.0269	0.9991	11.51 ± 0.08
Sulfate radical	*Y* = 2.2451x − 0.0146	0.9988	94.40 ± 1.36

**FIGURE 1:**
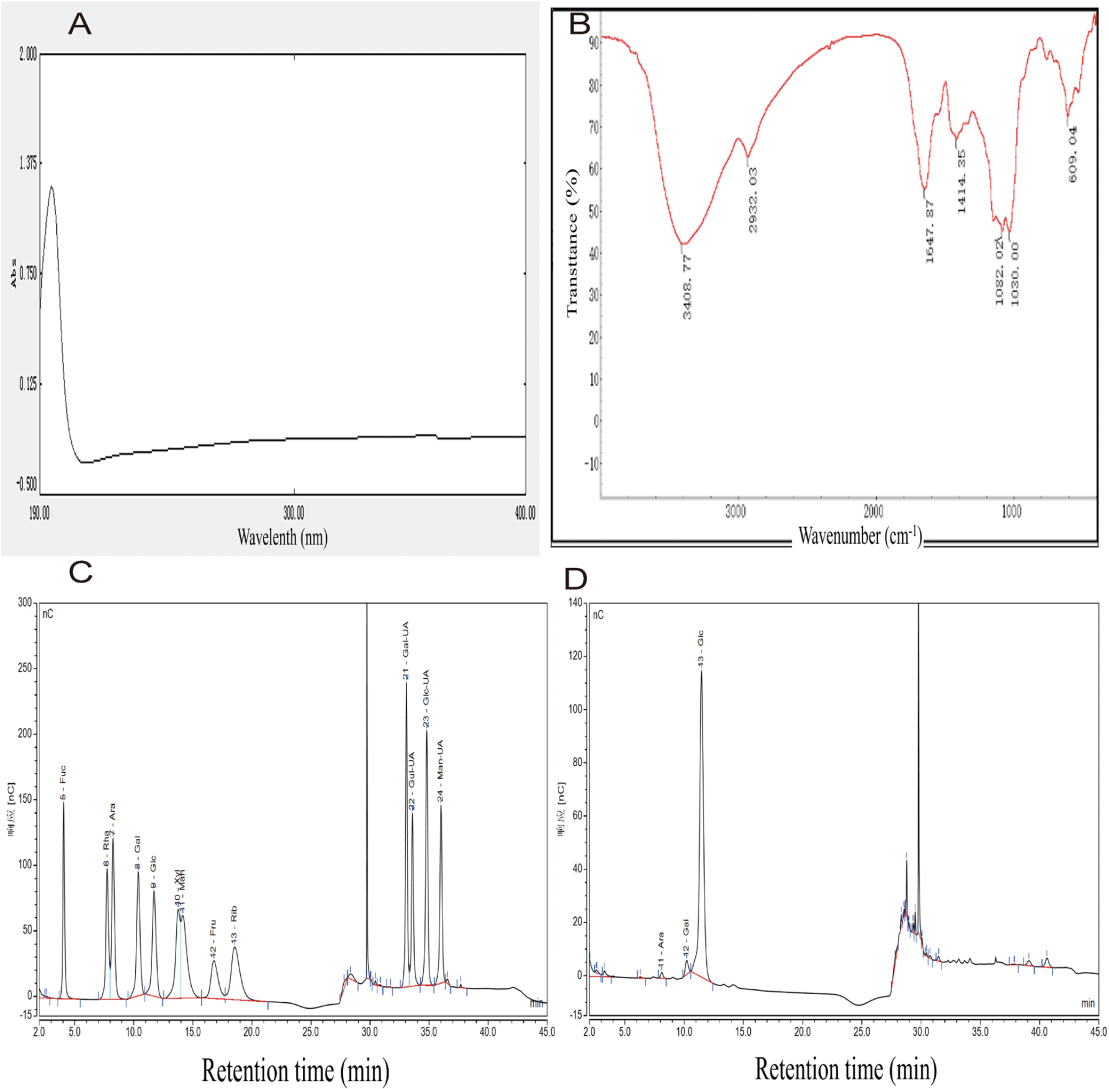
Spectral analysis of the PCP. (A) UV spectrum of PCP. (B) IR spectrum of PCP. (C, D) Monosaccharide composition of PCP.

To further accurately characterize PCP, UV and IR absorption spectroscopy were employed. The UV absorption spectrum exhibited a distinct peak at 195 nm (Figure 1A), which is characteristic of polysaccharides. Notably, no significant absorption peaks were observed at 260 and 280 nm, suggesting the presence of negligible quantities of nucleic acids and proteins in the sample. These findings indicate that the tested polysaccharide was extracted with high purity.

The IR spectrum of the sample provided further insights into its molecular structure (Figure 1B). The absorption band observed in the range of 3600–3200 cm^−1^ corresponds to the stretching vibration of ‐OH groups, which is a characteristic peak of carbohydrates. Specifically, the absorption peak at 3408.77 cm^−1^ is attributed to the stretching vibration of O‐H, a hallmark of carbohydrates. Additionally, the absorption peak at 2932.03 cm^−1^ is indicative of the stretching vibration of C‐H bonds, while the peak at 1082.02 cm^−1^ is associated with the stretching vibration of C‐O bonds.

Analysis of monosaccharide composition using 13 monosaccharides, which are fucose (Fuc), rhamnose (Rha), arabinose (Ara), galactose (Gal), glucose (Glc), xylose (Xyl), mannose (Man), fructose (Fru), ribose (Rib), galacturonic acid (Gal‐UA), glucuronic acid (Glc‐UA), mannuronic acid (Man‐UA), and guluronic acid (Gul‐UA) as monosaccharide standards.

As shown in Figure 1C,D, PCP is composed of arabinose, galactose, and glucose, with a molar ratio of arabinose: galactose: glucose of 1:2.08:64.3.

### 3.2 Effects of PCP on Urinary Protein and Serum Biomarker Levels in MRL/Lpr Mice

The concentrations of urinary protein, autoantibodies, inflammatory factors, renal function indicators, and complement factors in mice from different groups were measured using ELISA.

**FIGURE 2:**
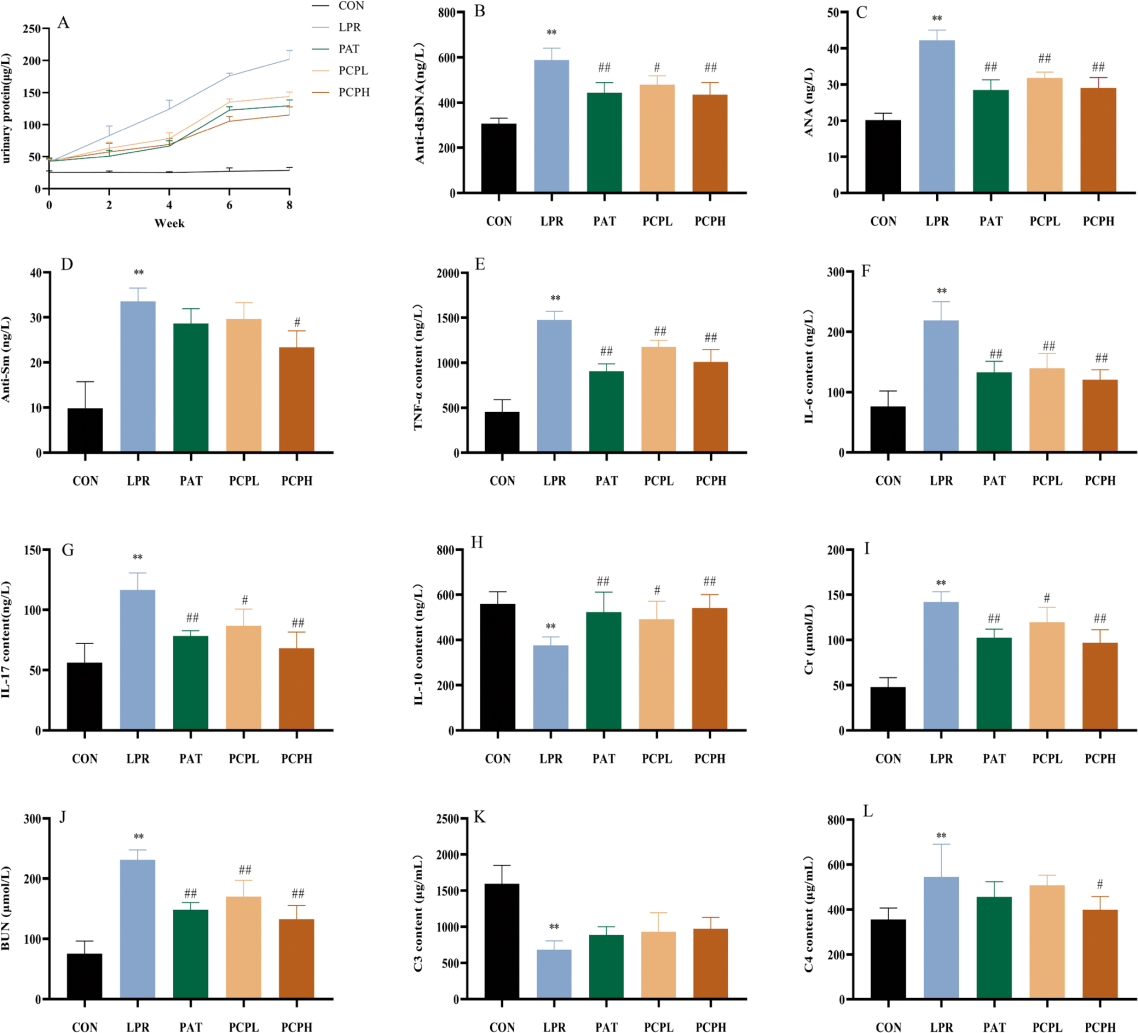
Effects of PCP on urinary protein, autoantibodies, inflammatory factors, renal function indicators and complement factors in MRL/lpr mice. (A) UP. (B) ds‐DNA. (C) ANA. (D) Sm. (E) TNF‐α. (F) IL‐6. (G) IL‐17. (H) IL‐10. (I) Cr. (J) BUN. (K) C3. (L) C4. Data were presented as mean ± SD (*n* = 6). Significant differences were indicated as *p < 0.05, **p < 0.01 versus CON. #*p* < 0.05, and ##*p* < 0.01 versus LPR.

As illustrated in Figure 2A, the urine protein (UP) content in the CON group remained stable over time, whereas in the LPR group, it progressively increased. Notably, PCP treatment was effective in reducing urine protein levels. Figure 2B–D show that compared to the CON group, the LPR group exhibited significantly elevated levels of anti‐dsDNA, ANA, and Sm antibodies. Specifically, in the LPR group, the concentrations of anti‐dsDNA, ANA, and Sm antibodies reached 588.19, 42.24, and 33.56 ng/L, respectively. Conversely, in the PCPL group, these levels were reduced to 479.86, 31.82, and 29.64 ng/L, respectively, while in the PCPH group, they were further reduced to 434.93, 29.01, and 23.40 ng/L, respectively. Figure 2E–H demonstrate that the LPR group had significantly higher levels of TNF‐α, IL‐6, and IL‐17 and a significantly lower level of IL‐10 compared to the CON group. Both PCPL and PCPH treatments were able to mitigate these inflammatory cytokine levels. Figure 2I,J indicate that serum creatinine (Cr) and blood urea nitrogen (BUN) levels were significantly higher in the LPR group than in the CON group but were significantly reduced in both the PCPL and PCPH groups. Figure 2K,L show that compared to the CON group, the LPR group had significantly lower levels of complement component 3 (C3) and higher levels of complement component 4 (C4). Both PCPL and PCPH treatments were able to normalize these complement levels. Importantly, all the aforementioned indicators in the PAT group also showed improvement.

### 3.3 Results of Kidney Tissue Histopathology Analysis and Immunofluorescence Findings

**FIGURE 3:**
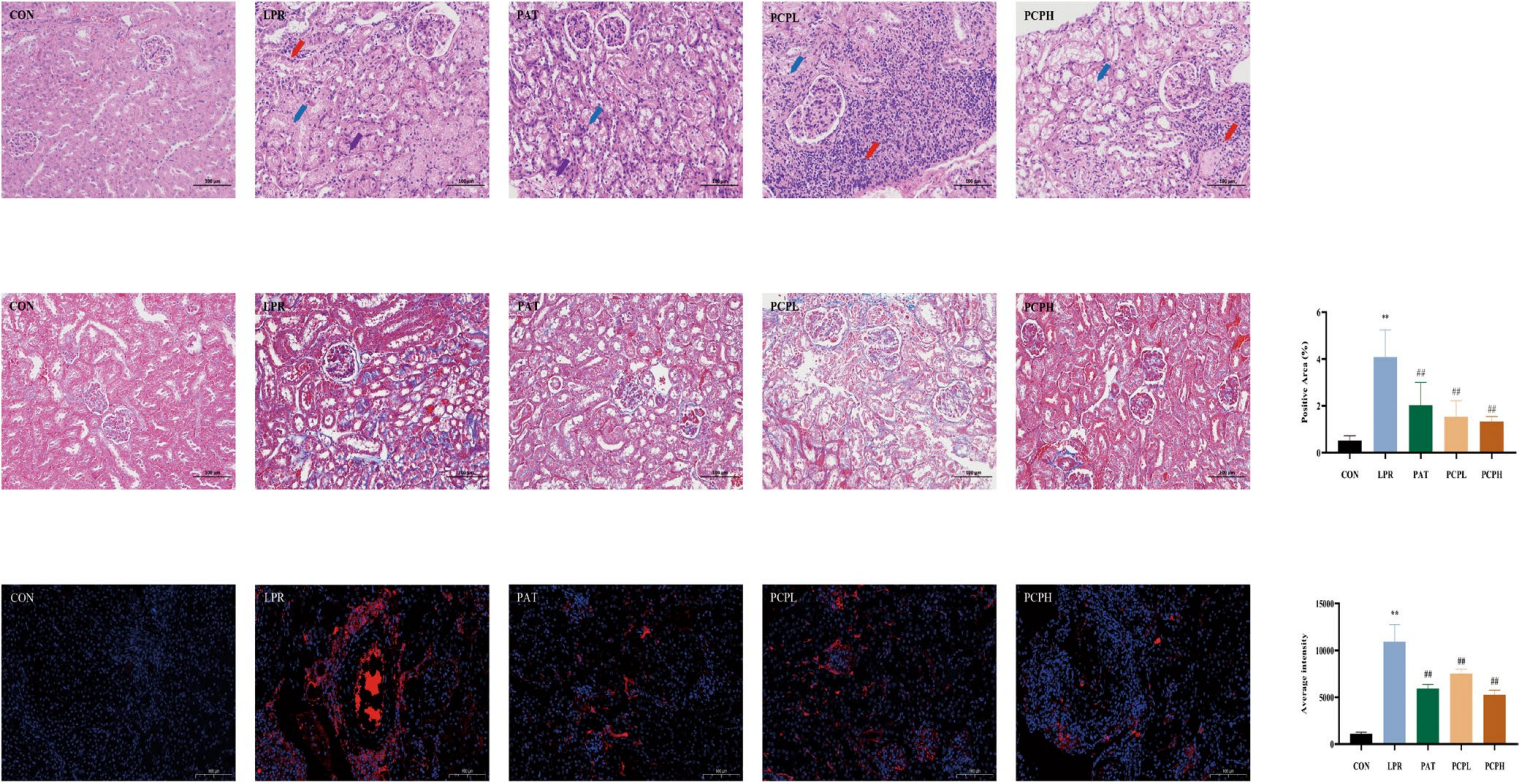
Results of kidney tissue histopathology analysis and immunofluorescence findings. (A) HE staining results. (B) Masson staining results and positive area ratio. (C) IgG Immunofluorescence staining and average intensity. Significant differences were indicated as *p < 0.05, **p < 0.01 versus CON. #*p* < 0.05, and ##*p* < 0.01 versus LPR.

The HE staining results, as shown in Figure 3A, indicate that in the CON group, the renal glomeruli showed no matrix hyperplasia, the renal tubular epithelial cells appeared round and plump, with a well‐organized brush border, and no notable abnormalities were detected. Additionally, the connective tissue between the urinary tubules, referred to as the renal interstitium, displayed no significant hyperplasia and lacked any evident inflammatory cell infiltration. In the LPR group, the renal glomeruli had a uniform number of cells and matrix; many renal tubular epithelial cells exhibited aqueous degeneration (indicated by blue arrows), with loose and pale cytoplasm, and occasional necrosis of renal tubular epithelial cells (indicated by purple arrows). Focal lymphocyte infiltration around vessels was noted in the interstitium (indicated by red arrows). Compared with the LPR group, the PAT group had a uniform number of glomerular cells and matrix, with fewer renal tubular epithelial cells showing aqueous degeneration (blue arrows), loose and pale cytoplasm, and occasional necrosis of renal tubular epithelial cells (purple arrows). In the PCPL group, the renal glomeruli had a uniform number of cells and matrix; many renal tubular epithelial cells exhibited aqueous degeneration (blue arrows), with loose and pale cytoplasm, and a small amount of perivascular lymphocyte focal infiltration was observed (red arrows). In the PCPH group, the renal glomeruli had a uniform number of cells and matrix; fewer renal tubular epithelial cells showed aqueous degeneration (blue arrows), with loose and pale cytoplasm, and rare perivascular lymphocyte focal infiltration was observed in the interstitium (red arrows).

The MASSON staining results, as shown in Figure 3B, indicate that compared to the CON group, the LPR group had a significant increase in the positive area ratio, which was 4.08% (*p* < 0.01). The PAT group positive area ratio was 2.02% (*p* < 0.05), the PCPL group positive area ratio was 1.54% (*p* < 0.05), and the PCPH group positive area ratio was 1.33% (*p* < 0.01). The IgG immunofluorescence results, as shown in Figure 3C, demonstrate that compared to the CON group, the LPR group had a significant increase in fluorescence intensity (*p* < 0.01). Under the intervention of PAT and PCP, the fluorescence intensity decreased (*p* < 0.01), indicating that PCPH has good pharmacological activity, providing better protection for the kidneys and promoting disease recovery. Therefore, in subsequent gut microbiota and metabolomics analyses, the pathways of action for PCPH were investigated.

### 3.4 Effects of PCP on MRL/Lpr Mice Gut Microbiota Dysbiosis

#### 3.4.1 Influence on Alpha and Beta Diversity

The Chao1 index serves as a metric for species' richness, which denotes the count of different species present in a given environment. On the other hand, the Shannon index is employed to quantify species diversity, a measure that is contingent upon both the species richness and the evenness of the species distribution within the community. When species richness is held constant, an increased evenness among species in a community corresponds to a higher level of diversity within that community. Elevated values for both the Chao1 and Shannon indices suggest a greater degree of species diversity within the sample being studied.

**FIGURE 4:**
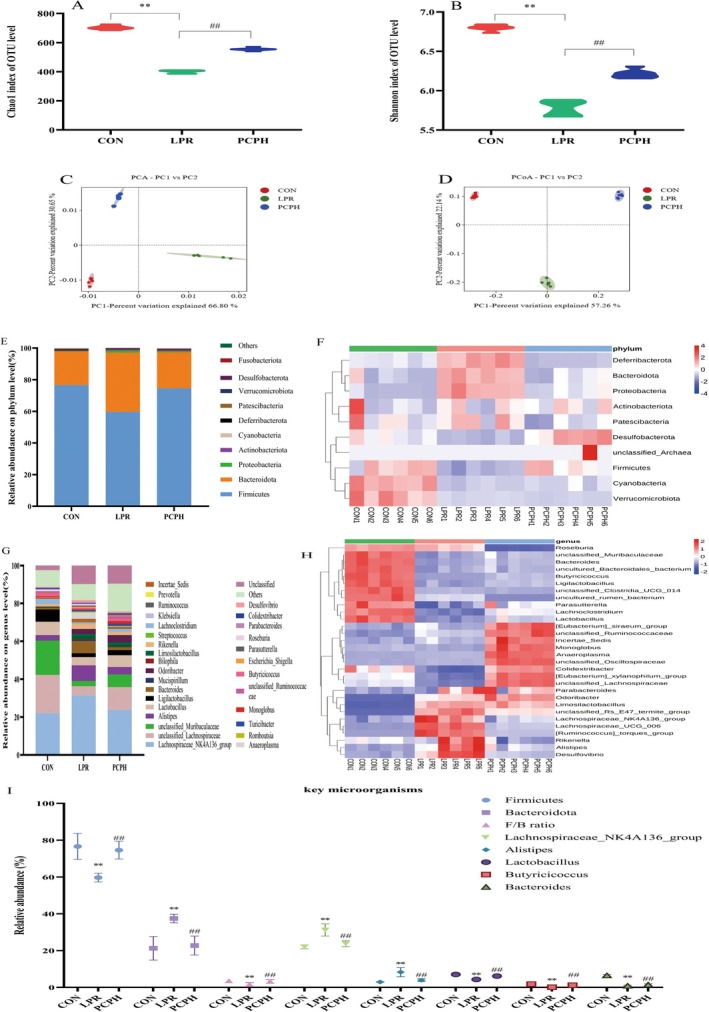
PCP modulated the gut microbiota diversity and composition in MRL/lpr mice. (A) Chao1 indices. (B) Shannon indices. (C) PCA analysis at the OTU level. (D) PCoA analysis at the OTU level. (E) Relative abundance of microbiota at phylum level (top 10). (F) Heatmap of microbiota at phylum level (top 10). (G) Relative abundance of microbiota at genus level (top 30). (H) Heatmap of microbiota at genus level (top 30). (I) Analysis of key microorganisms. Data were presented as mean ± SD (*n* = 6). Significant differences were indicated as *p < 0.05, **p < 0.01 versus CON; #*p* < 0.05, and ##*p* < 0.01 versus LPR. The significance of intergroup differences in Chao1 and Shannon indices was calculated using the one‐way Kruskal‐Wallis test (a non‐parametric ANOVA), with post hoc comparisons performed using Dunn's test. The significance of intergroup differences in PCA and PCoA analyses was determined by PERMANOVA (based on Bray‐Curtis distance). For key microbial analyses, intergroup significance was assessed using the Kruskal‐Wallis test, followed by pairwise post hoc comparisons with Dunn's test, adjusted for false discovery rate (FDR). A *p*‐value < 0.05 was considered statistically significant.

As illustrated in Figure 4A,B, the Chao1 and Shannon indices for the LPR group were markedly lower than those for the CON group (*p* < 0.01), signifying a decrease in both species richness and diversity within the LPR group. Conversely, PCPH treatment was found to partially restore species richness and diversity when compared to the LPR group (*p* < 0.01).

Furthermore, the results of PCA and PCoA analyses, as depicted in Figure 4C,D, highlighted significant discrepancies in community composition among the CON group and the LPR and PCPH groups. These findings indicate that PCPH has a discernible impact on the composition of the gut microbiota in mice.

#### 3.4.2 Influence on Gut Microbiota Composition

After analyzing the differences in α‐diversity and β‐diversity of the gut microbial communities, we further investigated the changes in the relative abundance of bacterial taxa. As shown in Figure 4E,F, at the phylum level, the dominant phylum of the three groups were *Firmicutes*, *Bacteroidota*, *Proteobacteria*, *Deferribacterota*, *Patescibacteria* and *Desulfobacterota*. In the CON group, they accounted for 76.62%, 21.25%, 0.27%, 0.59%, 0.36% and 0.31%, respectively. They also accounted for 59.69%, 37.45%, 1.21%, 0.12%, 0.17% and 0.91% in the LPR group, respectively, and 74.60%, 22.76%, 0.53%, 0.23%, 0.31% and 0.71% in the PCPH group, respectively. It can be seen that the proportion of the dominant phylum in the LPR group changed compared with the CON group, and the intervention of PCPH could reverse these changes to a certain extent. Moreover, we found that the gut microbiota of the LPR group showed a decrease in *Firmicutes* and an increase in *Bacteroidota* at the phylum level, resulting in a lower *Firmicutes* to *Bacteroidota* ratio (F/B ratio), which is consistent with the findings of existing studies [17]. PCPH treatment was able to revert the relative abundance of *Firmicutes* and *Bacteroidota*, promoting the normalization of the F/B ratio.

At the same time, we also performed a genus‐level analysis of the composition of the three groups of microbiotas. According to the analysis of dominant species in Figure 4G–I, we can conclude that the dominant species among the three groups differ. The proportion of the same species in each group is also different. Further differences in species composition were revealed through group‐to‐group comparative analysis. The proportion of *Lachnospiraceae* NK4A136 group in the CON group was 22.08%, which was significantly lower than the 31.36% in the LPR group. After PCPH treatment, the proportion of *Lachnospiraceae* NK4A136 group was 23.81%. The proportion of *Alistipes* in the CON group was 2.93%, which was significantly lower than the 8.30% in the LPR group. After PCPH treatment, the proportion of *Alistipes* was 3.93%. The proportion of *Lactobacillus* in the CON group was 7.05%, which was significantly higher than the 4.35% in the LPR group, and the proportion of *Lactobacillus* in the PCPH group was 6.18%. The proportion of *Bacteroides* in the CON group was 6.48%, which significantly decreased to 0.86% in the LPR group, and the proportion of *Bacteroides* in the PCPH group was 1.50%. The proportion of *Butyricicoccus* in the CON group was 1.80%, which was significantly reduced to 0.10% in the LPR group, and the proportion of *Butyricicoccus* in the PCPH group was 1.18%. In summary, significant differences exist among the three groups regarding gut microbiota composition, dominant species, and abundance, which may play a crucial role in disease progression and drug pharmacological mechanisms.

**FIGURE 5:**
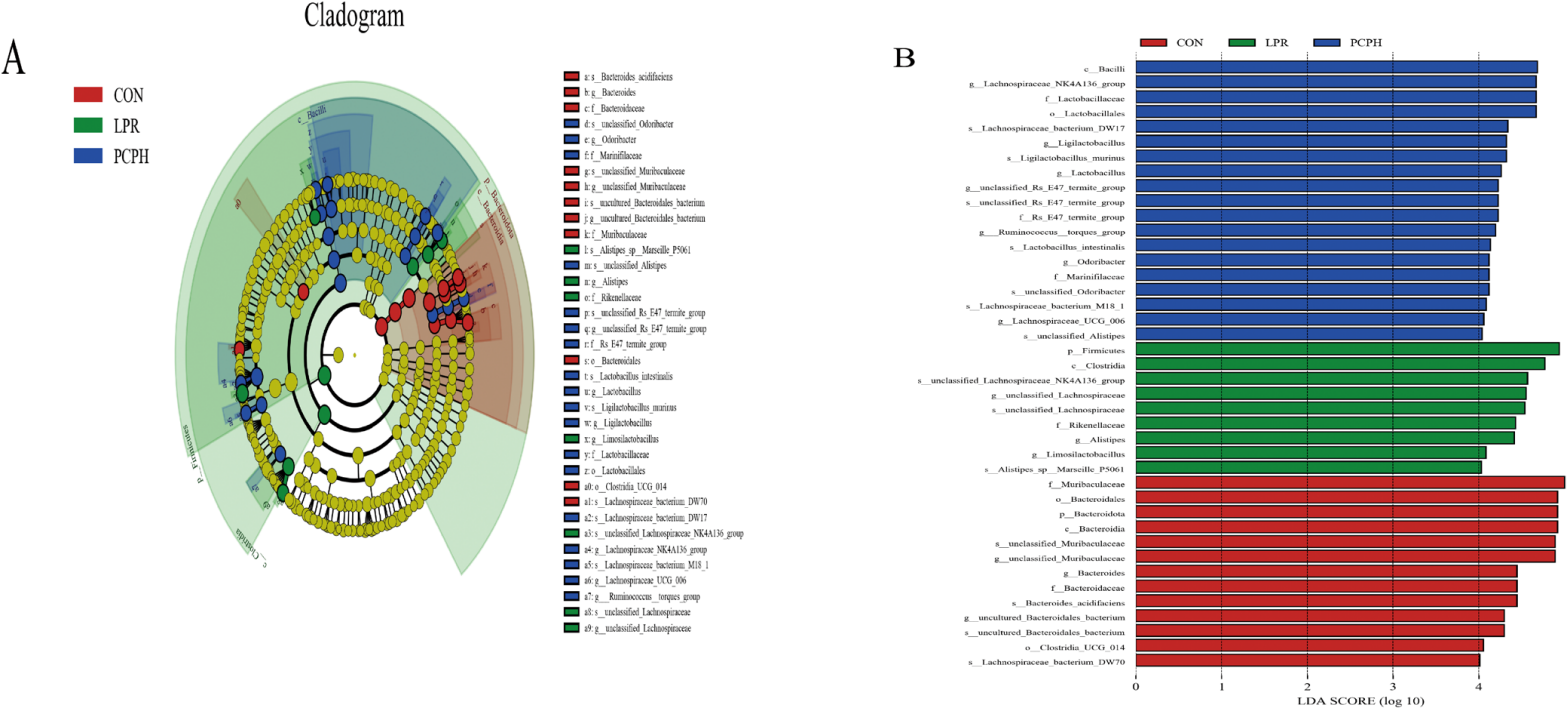
Effects of PCP on gut microbiota in MRL/lpr mice. (A) LEfSe taxonomic cladogram (LDA > 4). (B) LDA score (LDA > 4). In LEfSe analysis, significance was evaluated using the Kruskal‐Wallis test (*p* < 0.05) to identify differentially abundant taxa, with an LDA score > 4 considered biologically meaningful.

Figure 5A presents a cladogram constructed using Linear Discriminant Analysis (LDA) Effect Size (LEfSe), designed to visually compare microbial taxa exhibiting significant differences among the CON, LPR, and PCPH groups. In the diagram, each node represents a taxonomic level from phylum to genus, with the size of the node indicating the relative abundance of that taxonomic unit. Nodes of different colors signify taxa that are significantly enriched in their respective groups (red for the CON group, green for the LPR group, and blue for the PCPH group). The branches radiating from the center depict the evolutionary relationships among the various microbial taxa.

Figure 5B, in the form of a bar chart, displays the LDA scores for the microbial taxa that showed significant differences (LDA > 4). The length of each bar represents the magnitude of the effect size (LDA score) for that taxonomic unit among the different groups. The chart clearly lists the key microbial taxa significantly enriched in each group (CON, LPR, and PCPH) from the phylum to the genus level, along with their respective LDA scores. At the phylum and genus levels, *Bacteroidota*, *Uncultured Bacteroidales bacterium*, *Bacteroides*, and *unclassified Muribaculaceae* were enriched in the CON group. *Firmicutes*, *unclassified Lachnospiraceae*, *Limosilactobacillus*, and *Alistipes* were enriched in the LPR group. *Lachnospiraceae UCG 006*, *Odoribacter*, *Ruminococcustorques* group, *unclassified Rs E47 termite group, Ligilactobacillus*, and *Lactobacillus* were enriched in the PCPH group.

### 3.5 Correlation Analysis Between Gut Microbiota and LN‐Related Indicators

To further elucidate the association between gut microbiota and LN‐related indicators, we performed Spearman correlation analysis; the correlation was considered statistically significant with a correlation coefficient (|*p*| > 0.5).

**FIGURE 6:**
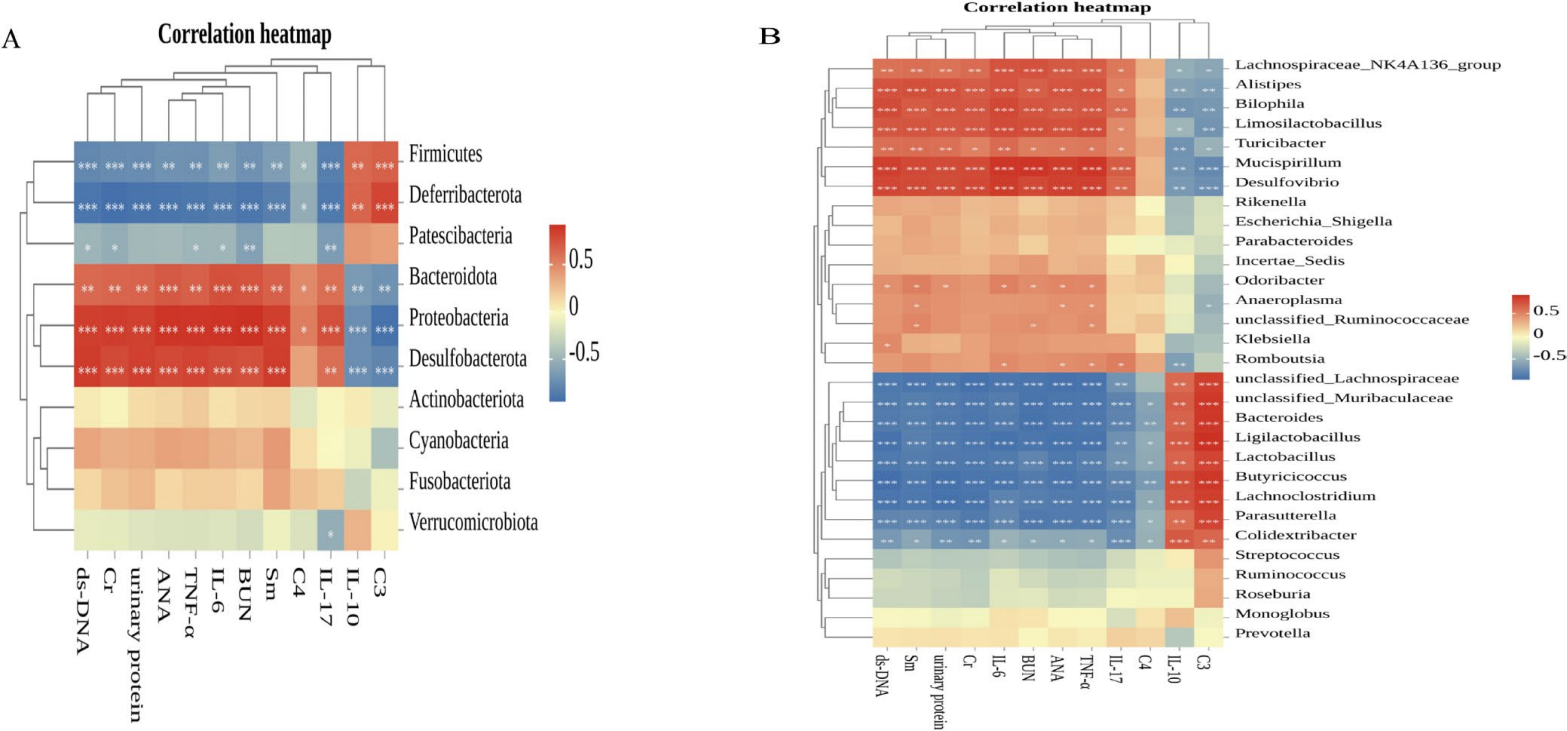
Correlation analysis between gut microbiota and LN‐related indicators. (A) The heatmap of Spearman correlation analysis between differential phylum level (top 10) and LN‐related indicators. (B) The heatmap of Spearman correlation analysis between differential genus level (top 30) and LN‐related indicators. Different colors represent the value of the correlation coefficient; red indicates positive correlation and blue indicates negative correlation. *p < 0.05, **p < 0.01, ***p < 0.001. The significance of correlations was calculated based on Spearman's rank test, where |*ρ*| > 0.5 and *p* < 0.05 (FDR corrected) were considered significant.

As shown in Figure 6A, at the phylum level, *Firmicutes* showed significant negative correlations with urinary protein, ds‐DNA, ANA, Sm, TNF‐α, IL‐6, IL‐17, BUN, Cr, and C4. Firmicutes showed significant positive correlations with C3 and IL‐10. *Bacteroidota* showed significant positive correlations with urinary protein, ds‐DNA, ANA, Sm, TNF‐α, IL‐6, IL‐17, BUN, Cr, and C4. *Bacteroidota* showed significant negative correlations with C3 and IL‐10. Additionally, *Proteobacteria*, *Deferribacterota*, and *Desulfobacterota* also exhibited strong correlations with these indicators.

As shown in Figure 6B, at the genus level, *Lachnospiraceae* NK4A136 group showed significant positive correlations with urinary protein, ds‐DNA, ANA, Sm, TNF‐α, IL‐6, IL‐17, BUN, and Cr. *Lachnospiraceae* NK4A136 group showed significant negative correlations with C3 and IL‐10. *Alistipes* showed significant positive correlations with urinary protein, ds‐DNA, ANA, Sm, TNF‐α, IL‐6, IL‐17, BUN, and Cr. *Alistipes* showed significant negative correlations with C3 and IL‐10. *Lactobacillus, Butyricicoccus*, and *Bacteroides* showed significant negative correlations with urinary protein, ds‐DNA, ANA, Sm, TNF‐α, IL‐6, IL‐17, BUN, Cr, and C4. *Lactobacillus, Butyricicoccus*, and *Bacteroides* showed significant positive correlations with C3 and IL‐10. Many other genera also showed strong correlations with these indicators.

### 3.6 PCP Improved the Serum Metabolic Profile of MRL/Lpr Mice

**FIGURE 7:**
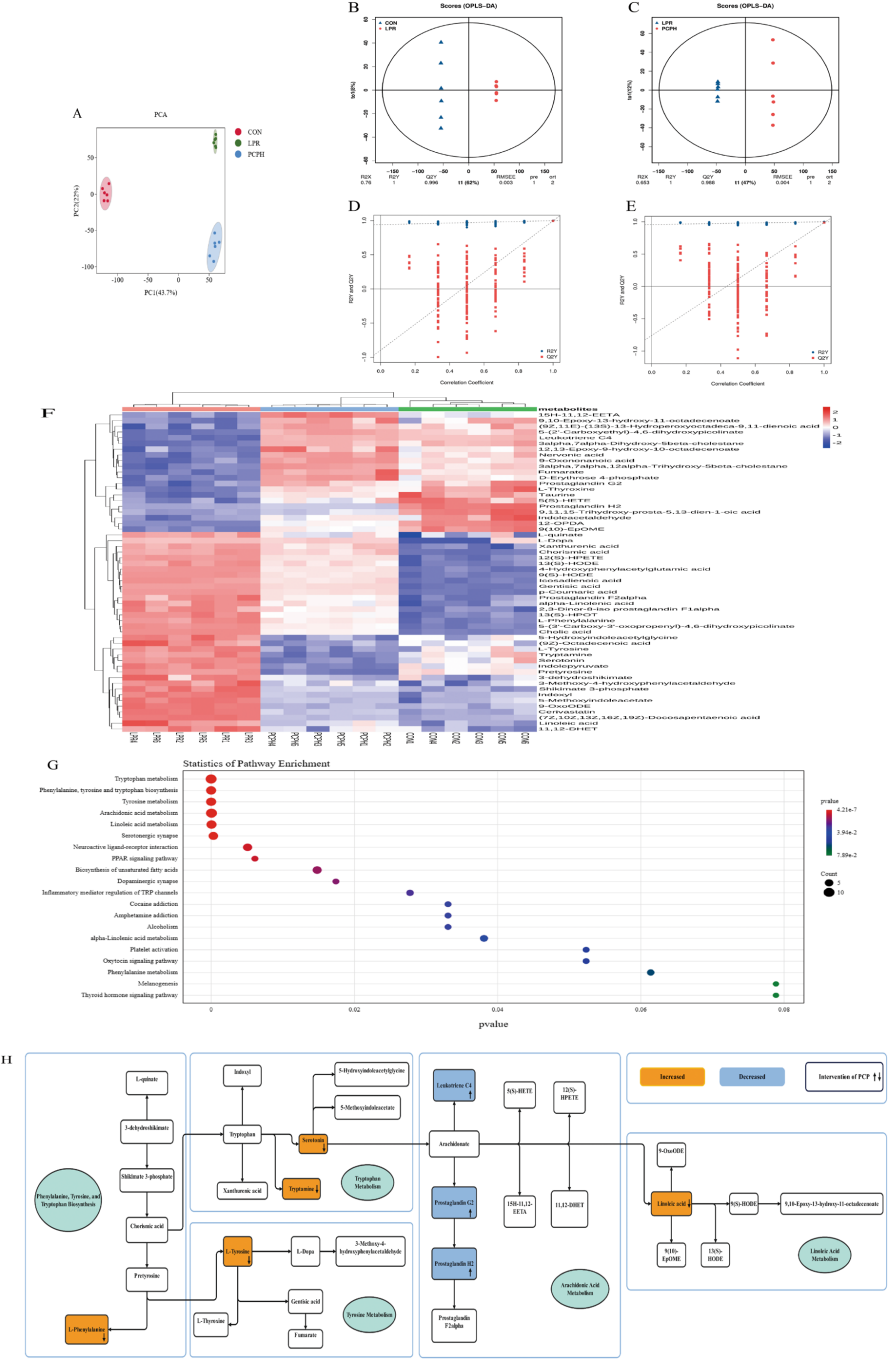
Effects of PCPH on the serum metabolites of MRL/lpr mice. (A) PCA. (B) OPLS‐DA between the CON and the LPR. (C) OPLS‐DA between the LPR and the PCPH. (D) RPT between the CON and the LPR. (E) RPT between the LPR and the PCPH. (F) Heatmap of significantly differential metabolites (FC > 1, VIP > 1.0, *p* < 0.01) among the CON group, LPR group, and PCPH group. (G) KEGG metabolic pathways enrichment analysis. The color of the point was *p*‐value, and the redder, the more significant enrichment. The size of the spot represented the number of different metabolites enriched. (H) The potential mechanism of PCP in treating LN. The color of the box represents the changing trend of the LPR group and the CON group, in which yellow represents promotion, and blue represents inhibition. The arrow on the right of the metabolite in the box indicates the trend of PCPH and the LPR group.

In this study, non‐targeted metabolomics techniques were used to detect serum metabolites. As shown in Figure 7A, the PCA score plot displayed tight clustering of samples between groups, indicating stable analysis conditions and good repeatability of the detection process. Furthermore, clear separation of serum metabolites is observed among the CON, LPR, and PCPH groups. The OPLS‐DA model distinguished between the CON group and LPR group (Figure 7B) with cumulative values of *R* ^2^Y and *Q* ^2^Y of 1 and 0.996, respectively, elucidating the stability and reliability of the model. The cumulative values of *R* ^2^Y and *Q* ^2^Y for the LPR group and PCPH group (Figure 7C) were 1 and 0.988, respectively, indicating the effectiveness of PCPH treatment. Additionally, the accuracy of the model was evaluated using the RPT method. As shown in Figure 7D,E the OPLS‐DA model revealed good precision.

By using the criteria of Fold Change (FC) > 1, variable importance in projection (VIP) > 1 and *p* < 0.01, we screened for differential metabolites from the CON group, LPR group, and PCPH group. We detected 56 candidate serum metabolites to be metabolic biomarkers that are mainly associated with amino acid, organic acid, and lipid. Compared with the CON group, 35 metabolites in the LPR group were up‐regulated, whereas 21 were down‐regulated. All potential biomarkers after PCPH administration intervention tend to return to the levels of the CON group (Additional file 1, Table S1). The metabolite heatmap is shown in Figure 7F.

Through KEGG enrichment analysis, these differential metabolites were mapped to specific pathways to determine the impact of PCPH on disease metabolic pathways. As shown in Figure 7G, the administration of PCPH significantly altered 6 metabolic pathways, including Tryptophan metabolism, Phenylalanine, tyrosine and tryptophan biosynthesis, Tyrosine metabolism, Arachidonic acid metabolism, Linoleic acid metabolism, and Serotonergic synapse. By constructing a metabolic pathway map, we have delineated the interconnected impacts of PCPH treatment on the Tryptophan metabolism, Phenylalanine, tyrosine and tryptophan biosynthesis, Tyrosine metabolism, Arachidonic acid metabolism, and Linoleic acid metabolism pathways. As depicted in Figure 7I, key metabolites within each pathway have been annotated, thereby uncovering the potential regulatory mechanisms of PCPH on these metabolic pathways. This visualization provides a foundational reference for further investigation into the role of PCPH in the progression of LN.

### 3.7 Correlation Analysis Between Gut Microbiota and Metabolites

**FIGURE 8:**
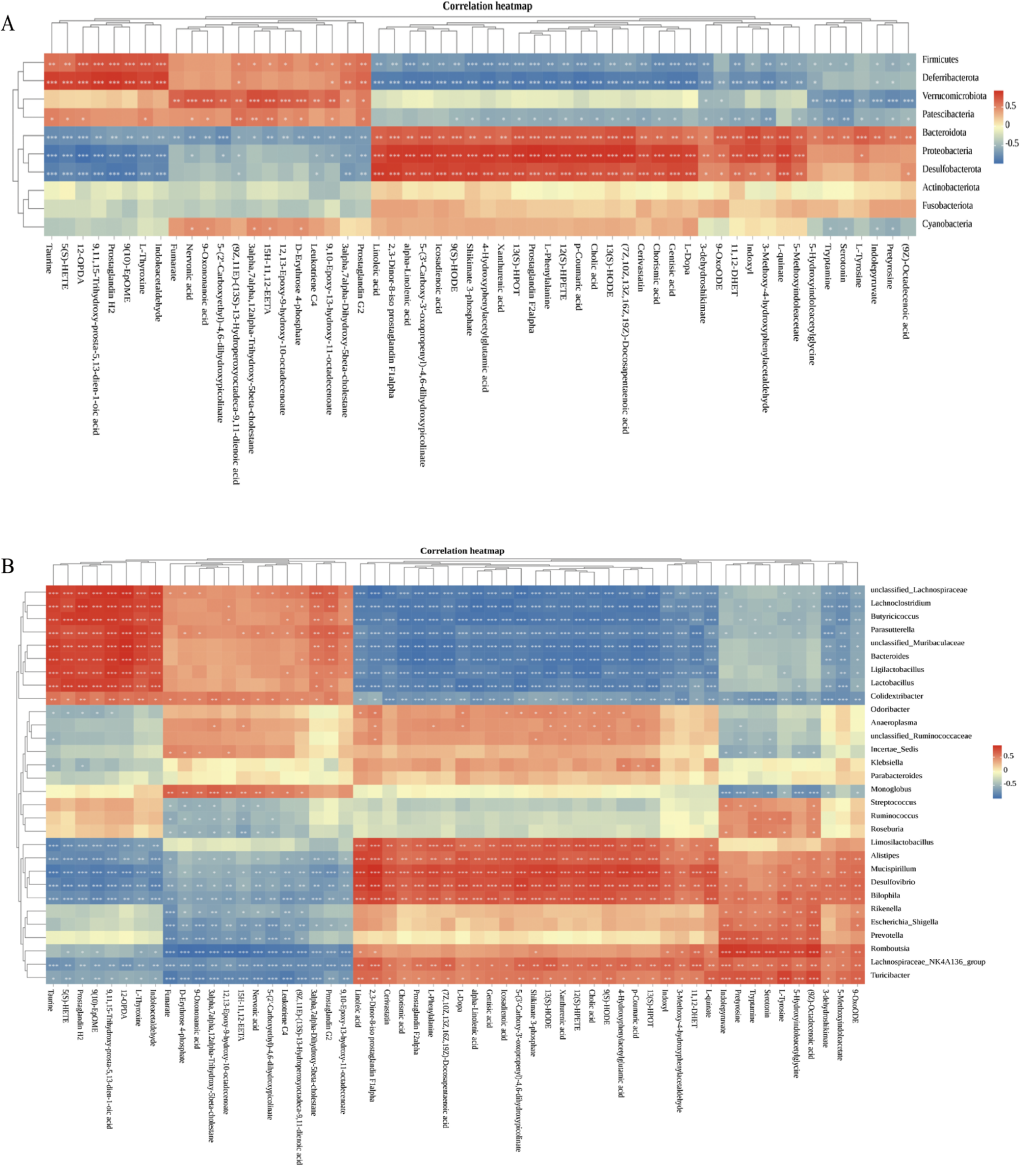
Correlation analysis between gut microbiota and metabolites. (A) The heatmap of Spearman's correlation analysis between differential phylum level (top 10) and metabolites. (B) The heatmap of Spearman's correlation analysis between differential genus level (top 30) and metabolites. Different colors represent the value of correlation coefficient; red indicates positive correlation and blue indicates negative correlation. *p < 0.05, **p < 0.01, ***p < 0.001. The significance of correlations was calculated based on Spearman's rank test, where |*ρ*| > 0.5 and *p* < 0.05 (FDR corrected) were considered significant.

Correlation heat maps illustrated the relationships between differential metabolites and gut microbiota (|*p*| > 0.5), highlighting the functional link between microbiota composition and body metabolism. As shown in Figure 8A, *Firmicutes* showed significant positive correlations with Leukotriene C4, Prostaglandin G2, and Prostaglandin H2, and negatively correlated with Linoleic acid, L‐Phenylalanine, Serotonin, and Tryptamine. *Bacteroidota* showed significant positive correlations with Linoleic acid, L‐Phenylalanine, Serotonin, Tryptamine, and L‐Tyrosine. *Bacteroidota* showed significant negative correlation with Leukotriene C4, Prostaglandin G2, and Prostaglandin H2. Additionally, other phyla, such as *Proteobacteria*, *Deferribacterota*, and *Desulfobacterota* also demonstrated significant correlations with various metabolites.

As shown in Figure 8B, at the genus level, the *Lachnospiraceae* NK4A136 group showed significant positive correlations with Linoleic acid, L‐Phenylalanine, Tryptamine, L‐Tyrosine, and Serotonin, and negatively correlated with Leukotriene C4, Prostaglandin G2, and Prostaglandin H2. *Alistipes* showed significant positive correlations with Linoleic acid and negatively correlated with Leukotriene C4, Prostaglandin G2, and Prostaglandin H2. *Butyricicoccus* showed significant positive correlations with Leukotriene C4, Prostaglandin G2, and Prostaglandin H2, and negatively correlated with Linoleic acid, L‐Phenylalanine, Tryptamine, and L‐Tyrosine. *Bacteroides* showed significant positive correlations with Prostaglandin G2 and Prostaglandin H2, and negatively correlated with Linoleic acid and L‐Phenylalanine. *Lactobacillus* showed significant positive correlations with Prostaglandin G2 and Prostaglandin H2, and negatively correlated with Linoleic acid and L‐Phenylalanine. Many other genera also showed strong correlations with metabolites. The results indicate a close relationship between metabolic products and the gut microbiota. PCP effectively alleviates the symptoms of LN in mice by regulating the interaction between these two factors.

## 4 Discussion

SLE is characterized by loss of tolerance against nuclear and cytoplasmic self‐antigens in genetically predisposed individuals, often after exposure to certain environmental triggers. Inappropriate activation of innate immunity sets in motion a cascade of immune reactions, with generation of autoreactive B cells, production of a broad spectrum of autoantibodies, and subsequent deposition of immune complexes in various organs, ensuing in tissue inflammation and damage (Anders and Rovin 2016; Anders et al. 2020). SLE is a chronic disease characterized by flares of activity alternating with periods of quiescence, but with relentless accrual of irreversible organ damage if disease activity fails to be quenched. LN is one of the most severe manifestations, affecting on average 35%–60% of patients with SLE (Mejia‐Vilet et al. 2022). Nevertheless, a significant proportion of patients with LN do not respond adequately to initial therapy and/or subsequently develop flares of activity and are at risk of progression to chronic kidney disease and ultimately kidney failure (Moroni et al. 2020; Parikh et al. 2014). Therefore, exploring innovative treatment approaches and identifying novel therapeutic agents are of paramount importance in enhancing the clinical outcomes of LN.

With the continuous exploration of fungal components, we have found that PCP shows potential in the treatment of LN. As a type of fungal polysaccharide, the PCP can regulate gut microbiota. Gut microbiota plays an essential role in the homeostasis of the human body environment and is known as the natural barrier of the human body. It is also involved in the energy conversion and metabolism of the body and is associated with type 1 diabetes (Knip and Siljander 2016), rheumatoid arthritis (de Van Wiele et al. 2016), and LN (Opazo et al. 2018). Moreover, gut microbiota is mainly involved in the progression of LN through some metabolites, such as short‐chain fatty acids (SCFAs), endotoxin, and bile acids (BAs), etc. (Chen et al. 2016). Metabolomics can comprehensively and intuitively study various metabolic dynamic changes of organisms under the influence of physiology, pathology, and drugs, and combined with the gut microbiome, can discover potential metabolic pathway changes that interact between gut microbiota and the body. This study systematically investigated the effects of PCP on MRL/lpr mice from various aspects including urinary protein, autoantibodies, inflammatory cytokines, and renal function indicators. Kidney lesions were observed using HE staining, Masson's staining, and IgG immunofluorescence. Furthermore, gut microbiome omics and UHPLC–MS untargeted metabolomics were used to analyze the mechanism of PCP intervention in MRL/lpr mice.

In this study, we used the most commonly used animal model for LN research, MRL/lpr mice, which are genetically susceptible to SLE due to a mutation in the Fas protein coding gene. These mice spontaneously produce autoantibodies and develop lupus‐like symptoms (Watanabe‐Fukunaga et al. 1992). We intervened with low‐dose PCP and high‐dose PCP in MRL/lpr mice and found that PCP effectively restored urinary protein levels in MRL/lpr model mice. Additionally, we observed increased levels of autoantibodies (ds‐DNA, ANA, and Sm) in MRL/lpr mice, which are biomarkers for LN and rise during its progression [27–29]. Simultaneously, levels of pro‐inflammatory cytokines (TNF‐α, IL‐6, and IL‐17) increased, while the level of the anti‐inflammatory cytokine IL‐10 decreased, with these factors being used for the diagnosis and monitoring of LN [30–33]. BUN, Cr, C3, and C4 are indicators that can be used to assess renal function status. After PCP intervention, the levels of autoantibodies, inflammatory cytokines, and renal function indicators in MRL/lpr mice were significantly improved. HE staining results showed that after PCP treatment, the degenerative damage of renal tubular epithelial cells was significantly improved, indicating that PCP helps to restore the kidney's filtering and excretory functions. Masson staining further revealed that PCP treatment effectively reduced kidney fibrosis. The results of IgG immunofluorescence detection indicated that PCP intervention reduced the deposition of immune complexes. All results indicate that PCP can improve urine protein, autoimmune antibodies, inflammatory factors, renal function levels, and protect the kidneys, with the most significant impact observed at high doses of PCP.

### 4.1 Gut Microbiome Analysis

High population richness, diversity, and stable gut microbiota composition are hallmarks of healthy gut microbial communities (Ahrodia et al. 2022). Clinical studies have found that the abundance and diversity of gut microbiota are significantly reduced in patients with diagnosed LN compared with healthy controls, and such changes may be caused by changes in diet and the body's response (Manfredo Vieira et al. 2018). In this study, we also observed the same phenomenon. The Chao1 and Shannon indexes of microbiota in MRL/lpr mice indicated that the abundance and diversity of gut microbiota were significantly reduced, suggesting that microbiota health in MRL/lpr mice decreased.

Community analysis of the gut microbiota at the phylum level revealed that *Firmicutes* and *Bacteroidota* are the dominant groups. Comparing the CON and LPR groups, we observed a reduction in *Firmicutes* and a corresponding increase in *Bacteroidota* within the LPR group. This resulted in a lower *Firmicutes*‐to‐*Bacteroidota* (F/B) ratio in the LPR group. A lower F/B ratio may lead to an imbalance between regulatory T cells (Tregs) and Th17 cells, a phenomenon that could exacerbate existing intestinal inflammation conditions (López et al. 2016). In patients with LN, a decrease in the F/B ratio has also been observed, which has become an important characteristic of the gut ecological imbalance in LN patients (Hevia et al. 2014). Furthermore, experimental results reveal a significant correlation between the *Firmicutes*, the *Bacteroidota* and markers of inflammation, autoantibodies, and renal function. This indicates that PCP treatment effectively modulates the imbalance between *Firmicutes* and *Bacteroidota*, thereby contributing to the improvement of overall health. This could be one of the effective pathways through which PCP ameliorates LN.

At the genus level, an increase in the abundance of the *Lachnospiraceae* family, particularly the NK4A136 group, in the gut suggests a disruption of metabolic functions, which is often associated with the destruction of the intestinal barrier and systemic inflammation (Wang, Ma, et al. 2022). The microbial metabolites SCFAs, such as butyrate, and bile acids may activate renal local inflammatory pathways, such as NF‐κB, through the “gut‐kidney axis,” promoting the release of pro‐inflammatory factors like IL‐6 and TNF‐α, thereby triggering an inflammatory response (Wang et al. 2024). An increased abundance of *Alistipes* has been reported to be associated with disease activity in both inflammatory bowel disease and LN (Li et al. 2024). It may exacerbate the expression of local IL‐17 and TNF‐α in the kidneys by activating the TLR4/NF‐κB pathway through lipopolysaccharide (LPS). During the active phase of LN, the disease activity may induce *Alistipes* to shift its metabolic pathway towards pro‐inflammatory substances such as indole derivatives, further disrupting the Th17/Treg balance (Akhgar et al. 2023). In our study, the abundance of the *Lachnospiraceae* NK4A136 group and *Alistipes* significantly increased in the LPR group. This finding indicates that the LPR group is under inflammatory attack, further revealing the close connection between gut microbiota imbalance and LN. Concurrently, these two bacterial groups show a positive correlation with markers such as autoantibodies and inflammatory factors, suggesting that they might represent critical microbial communities involved in the pathogenesis of LN. PCP can inhibit the growth of the *Lachnospiraceae* NK4A136 group and *Alistipes*, thereby breaking the vicious cycle of “gut‐derived inflammation‐kidney damage.”

*Butyricicoccus* is an important genus of butyrate‐producing bacteria, and its metabolic product butyrate alleviates renal immune complex deposition by inhibiting B cell activation and the production of autoantibodies. Its reduction may lead to excessive activation of B cells, exacerbating autoantibody‐mediated kidney damage (Eeckhaut et al. 2013). In LN, butyrate also acts as an inhibitor of histone deacetylase (HDAC), improving the imbalance of T cell function through epigenetic regulation and reducing the accumulation of pro‐inflammatory metabolites such as trimethylamine N‐oxide (TMAO) (Lau et al. 2021). *Lactobacillus* effectively inhibits the colonization of pathogenic bacteria by secreting antimicrobial peptides, such as defensins, and upregulates the expression of intestinal tight junction proteins (such as ZO‐1), reducing endotoxin translocation and systemic inflammation (Yang et al. 2018). This strategy of enhancing barrier function through the modulation of tight junctions has also been validated in other natural products. For instance, a previous study by Xia et al. (2020) demonstrated that *Lycium* Berry Polysaccharides similarly improve intestinal barrier function by upregulating tight junction molecules. This highlights the pivotal role of this pathway in maintaining gut health and alleviating inflammation. Additionally, this bacterial species regulates the host immune response by influencing the tryptophan metabolic pathway, converting tryptophan into indole lactic acid (ILA) and indole‐3‐carbinol (I3C). These metabolites activate the aryl hydrocarbon receptor (AhR), thereby promoting the differentiation of regulatory T cells and inhibiting Th17‐mediated inflammatory responses (Wang, Zhao, et al. 2022). In this study, the increase of *Bacteroides* may further reinforce this process, as it can produce SCFAs that synergize with AhR signaling to enhance intestinal barrier function (Zhu et al. 2022). Additionally, correlation analysis indicates that *Butyricicoccus*, *Lactobacillus*, and *Bacteroides* are negatively associated with autoantibodies and pro‐inflammatory factors. PCP can restore the abundance of *Butyricicoccus*, *Lactobacillus*, and *Bacteroides*, thereby repairing the gut barrier function, exerting broad anti‐inflammatory and immunomodulatory effects that facilitate recovery from LN.

The LEfSe analysis further pinpointed specific microbial taxa that underwent significant changes in the disease state of the LPR group and following PCPH intervention. Notably, the LPR group showed significant enrichment of the phylum Firmicutes and genera including *Alistipes* and *Limosilactobacillus*. This was consistent with our observations of a decreased F/B ratio and increased abundance of *Alistipes* in the LPR group. The enrichment of *Alistipes* has been reported to be associated with the activity of inflammatory bowel disease and LN. It may exacerbate local renal inflammation by producing pro‐inflammatory substances like LPS, thereby activating the TLR4/NF‐κB pathway (Ma et al. 2024). The significance of *Limosilactobacillus* enrichment in lupus models, which often includes certain *Lactobacillus* members, requires further elucidation and may reflect a specific state of dysbiosis (Park et al. 2025).

In contrast, PCPH intervention led to significant enrichment of several genera with potentially beneficial functions, including the *Lachnospiraceae* NK4A136 group, *Lactobacillus*, *Odoribacter*, *Ruminococcaceae* UCG‐014, *Ligilactobacillus*, and *Lachnospiraceae* UCG 006. Among these, the enrichment of the *Lachnospiraceae* NK4A136 group and *Lactobacillus* was particularly crucial. Although the total abundance of the *Lachnospiraceae* NK4A136 group was elevated in the LPR group in our study, the LEfSe analysis revealed that PCPH intervention led to the significant enrichment of specific subgroups within this taxon (such as the *Lachnospiraceae* NK4A136 group itself as identified by the analysis). This suggests that PCPH may selectively promote subgroups with specific functions within this taxon. Many members of the Lachnospiraceae family are important butyrate‐producing bacteria. Butyrate, as a key SCFA, plays roles in enhancing gut barrier function, inhibiting inflammation, and regulating immunity (Yu et al. 2022). As a classic probiotic, the enrichment of *Lactobacillus* helps inhibit pathogenic bacteria colonization, maintain gut barrier integrity, and exert immunomodulatory effects by modulating tryptophan metabolism to activate the AhR (Long et al. 2025). *Odoribacter* and *Ruminococcaceae* UCG‐014 have also been reported to be associated with SCFA production and anti‐inflammatory effects (Zheng et al. 2025). PCPH intervention significantly modulates the structure of the gut microbiota, which is accompanied by the alleviation of systemic inflammation. Notably, complement C3 may play a pivotal bridging role in intestinal mucosal immunity. Furthermore, reports that saponin‐type natural compounds exert immunomodulatory effects by regulating complement C3 provide a useful analogy and supporting evidence for understanding the effects of PCPH. For instance, saponins isolated from *Radix polygalae* have been demonstrated to modulate the aging process and improve gut microbial diversity (Zeng et al. 2021). Although PCP is a polysaccharide and thus chemically distinct from saponins, both are naturally derived bioactive molecules. They may regulate C3 and its related pathways through a multi‐target approach, thereby offering superior intervention strategies for autoimmune diseases such as LN.

### 4.2 HPLC‐MS Untargeted Metabolomics Analysis

From UHPLC–MS untargeted metabolomics analysis, this study found that PCP treatment in MRL/lpr mice affected the tryptophan metabolism, phenylalanine, tyrosine and tryptophan biosynthesis, tyrosine metabolism, arachidonic acid metabolism and linoleic acid metabolism, which play a crucial role in immune regulation and inflammatory diseases. Linoleic acid is a precursor to arachidonic acid, which in the body is metabolized through the pathways of cyclooxygenase (COX) and lipoxygenase (LOX), respectively converting into prostaglandins and leukotrienes. If the level of linoleic acid rises, this may indicate an obstruction in the downstream metabolic pathways, which in turn leads to a reduction in the synthesis of prostaglandins and leukotrienes (Wang, Wu, et al. 2021). A decrease in leukotriene C4 levels is a compensatory anti‐inflammatory response during the progression of a disease, but this may exacerbate tissue damage, particularly having an adverse effect on the glomerular filtration function of the kidneys (Landgraf et al. 2014). Prostaglandin G2 and prostaglandin H2 play a dual role in regulating the inflammatory response, and their reduction may alter the renal microenvironment, decrease the permeability of renal blood vessels, or inhibit the invasion of immune cells (Gupta et al. 2015). PCP can significantly reduce levels of linoleic acid while markedly increasing the levels of leukotrienes and prostaglandins, thereby regulating linoleic acid metabolism and arachidonic acid metabolism. This contributes to restoring the balance of inflammation within the body, mitigating kidney damage, and alleviating the progression of the disease.

L‐Phenylalanine and L‐Tyrosine, as aromatic amino acids, their accumulation may indicate enhanced protein catabolism or impairment of liver metabolic function, which is a common phenomenon in chronic inflammatory diseases (Rohraff et al. 2019). The metabolism of tryptophan usually proceeds through the kynurenine pathway or the serotonin pathway (Fernstrom 2016). In LN, the kynurenine pathway may be inhibited, leading to a greater conversion of tryptophan into serotonin and tryptamine. Serotonin, by activating 5‐HT receptors, may exacerbate leakage in kidney vessels and promote the deposition of immune complexes. Additionally, an imbalance of the gut microbiota can lead to increased levels of tryptamine, which activates the aromatic hydrocarbon receptor (AhR), thereby promoting plasma cells to secrete autoantibodies, intensifying the immune system's attack on self‐tissues and resulting in a vicious cycle of inflammatory responses (Roveta et al. 2024). After PCP administration, L‐Phenylalanine, L‐Tyrosine, serotonin, and tryptamine all significantly decreased, thereby regulating tryptophan metabolism, phenylalanine, tyrosine and tryptophan biosynthesis, and tyrosine metabolism. Tryptophan metabolism, phenylalanine, tyrosine and tryptophan biosynthesis, tyrosine metabolism, linoleic acid metabolism, and arachidonic acid metabolism may be potential pathways through which PCP exerts its effects and warrant further investigation.

### 4.3 Correlation Between the Metabolites and Gut Microbiota

The relationship between gut microbiota and its host is reciprocal and symbiotic. Not only do the diet and metabolism of the body determine the composition and diversity of gut microorganisms, but gut microbiota and its bioactive metabolites have also been confirmed to regulate the bioenergy and metabolism of the host (Wahlström et al. 2016). Therefore, combined analysis of serum UHPLC–MS untargeted metabolomics and gut microbiome omics can obtain deeper biological information. We eventually found that *Firmicutes*, *Bacteroidota*, and LN regulated the serum differential metabolites. Consequently, these microorganisms contribute to macroscopic characteristics such as reducing autoantibodies, lowering inflammatory response, and restoring renal function in LN by establishing micro‐relationships with the body, such as amino acid metabolism, substance exchange, and biosynthesis. These microorganisms are also potential biological targets for the treatment of LN in the future. PCP, as an oral medicine with the same source of medicine and food, must directly affect gut microorganisms' metabolism and ecological environment in the digestive tract. Our study shows that PCP affected the proportion and abundance of *Lachnospiraceae* NK4A136 group, *Alistipes*, *Butyricicoccus*, *Bacteroides*, and *Lactobacillus* to modify changes in Tryptophan metabolism, Phenylalanine, Tyrosine and Tryptophan biosynthesis, Tyrosine metabolism, Arachidonic acid metabolism, and Linoleic acid metabolism pathways and alleviate the inflammatory response and renal dysfunction caused by autoimmune abnormalities. However, its mechanism of action needs further research to clarify its specific pharmacological action and determine its clinical significance.

## 5 Conclusions

This study showed that PCP effectively reduces autoantibody levels, alleviates inflammatory responses, and promotes the improvement of renal function in the treatment of MRL/lpr mice. Based on 16 s rDNA gut microbiome and UHPLC–MS untargeted metabolomics analysis, PCP can affect gut microbiota's abundance, diversity, and stability by affecting the abundance, diversity, and stability. It also regulates three phyla of *Firmicutes*, *Bacteroidota*, and five genera of *Lachnospiraceae* NK4A136 group, *Alistipes*, *Butyricicoccus*, *Bacteroides*, and *Lactobacillus*, thereby inducing changes in the tryptophan metabolism, phenylalanine, tyrosine and tryptophan biosynthesis, tyrosine metabolism, linoleic acid metabolism, and arachidonic acid metabolism pathways associated with LN. This process is the result of the synergistic action of various gut bacteria. Therefore, this study provides a new method to integrate gut microbiome and UHPLC–MS untargeted metabolomics to evaluate the pharmacodynamics and mechanism of PCP intervention in LN, which provides valuable ideas and insights for future research on the therapeutic application of fungal components in treating LN.

## Author Contributions

**Guoxin Ji:** writing – original draft (equal). **Cuicui Li:** conceptualization (equal). **Zhuangzhuang Yao:** visualization (equal). **Zhimeng Li:** formal analysis (equal). **Bo Yang:** methodology (equal). **Liming Hu:** software (equal). **Hang Yu:** software (equal). **Tongwei Jiang:** resources (equal). **Huan Wang:** writing – review and editing (equal).

## Funding

This work was supported by the Science and Technology Development Program Project of Jilin Province [grant number 20240303118NC].

## Conflicts of Interest

The authors declare no conflicts of interest.

## Supporting information


**Table S1:** Differential metabolites in mouse serum between groups. The *p*‐value CONVS.LPR < 0.01. The P‐value LPRVS.PCPH < 0.01.

## Data Availability

The data presented in this study can be made available by the corresponding author upon request.
