# Supplementary material for: Therapeutic Potential of Irpex lacteus Polysaccharides in Lupus Nephritis: Insights From Gut Microbiota and Metabolomics Analysis in MRL/Lpr Mice
Source: Food Sci Nutr. 2026 Jan 9;14(1):e71446. doi: 10.1002/fsn3.71446 (PMC12789650; doi:10.1002/fsn3.71446)
Supplement: Supplementary file 1 — Table S1: Differential metabolites in mouse serum between groups. The p‐value CONVS.LPR < 0.01. The P‐value LPRVS.PCPH < 0.01. [file FSN3-14-e71446-s001.docx]

**Table S1.** Differential metabolites in mouse serum between groups. The P-value CONVS.LPR < 0.01. The P-value LPRVS.PCPH < 0.01.

| Metabolite | Library ID | CONVS.LPR | LPRVS.PCPH |
| --- | --- | --- | --- |
| Taurine | HMDB0000251 | down | up |
| Linoleic acid | HMDB0000673 | up | down |
| Shikimate 3-phosphate | HMDB0301781 | up | down |
| Fumarate | HMDB0000131 | down | up |
| 5-Hydroxyindoleacetylglycine | HMDB0004185 | up | down |
| D-Erythrose 4-phosphate | HMDB0001321 | down | up |
| 3-dehydroshikimate | HMDB0304122 | up | down |
| Indolepyruvate | HMDB0060484 | up | down |
| Pretyrosine | HMDB0304809 | up | down |
| Cerivastatin | -- | up | down |
| Nervonic acid | HMDB0002368 | down | up |
| Chorismic acid | HMDB0012199 | up | down |
| Tryptamine | HMDB0000303 | up | down |
| 4-Hydroxyphenylacetylglutamic acid | HMDB0006061 | up | down |
| Indoxyl | HMDB0004094 | up | down |
| Xanthurenic acid | HMDB0000881 | up | down |
| L-Dopa | HMDB0000181 | up | down |
| Gentisic acid | HMDB0000152 | up | down |
| p-Coumaric acid | HMDB0002035 | up | down |
| 9-Oxononanoic acid | -- | down | up |
| 5-Methoxyindoleacetate | HMDB0004096 | up | down |
| Leukotriene C4 | HMDB0001198 | down | up |
| 13(S)-HPOT | HMDB0301803 | up | down |
| Prostaglandin H2 | HMDB0001381 | down | up |
| Prostaglandin F2alpha | HMDB0001139 | up | down |
| (7Z,10Z,13Z,16Z,19Z)-Docosapentaenoic acid | -- | up | down |
| 12(S)-HPETE | HMDB0004243 | up | down |
| alpha-Linolenic acid | HMDB0001388 | up | down |
| Icosadienoic acid | -- | up | down |
| 12-OPDA | HMDB0301804 | down | up |
| 13(S)-HODE | -- | up | down |
| (9Z,11E)-(13S)-13-Hydroperoxyoctadeca-9,11-dienoic acid | HMDB0003871 | down | up |
| 9,11,15-Trihydroxy-prosta-5,13-dien-1-oic acid | HMDB0005083 | down | up |
| 3alpha,7alpha,12alpha-Trihydroxy-5beta-cholestane | HMDB0001457 | down | up |
| (9Z)-Octadecenoic acid | HMDB0000207 | up | down |
| 3alpha,7alpha-Dihydroxy-5beta-cholestane | HMDB0006893 | down | up |
| 9,10-Epoxy-13-hydroxy-11-octadecenoate | -- | down | up |
| L-quinate | HMDB0304404 | up | down |
| Serotonin | HMDB0000259 | up | down |
| L-Phenylalanine | HMDB0000159 | up | down |
| Prostaglandin G2 | HMDB0003235 | down | up |
| 3-Methoxy-4-hydroxyphenylacetaldehyde | HMDB0005175 | up | down |
| 5-(3'-Carboxy-3'-oxopropenyl)-4,6-dihydroxypicolinate | -- | up | down |
| 15H-11,12-EETA | HMDB0005050 | down | up |
| L-Thyroxine | HMDB0000248 | down | up |
| Cholic acid | HMDB0000619 | up | down |
| 11,12-DHET | HMDB0002314 | up | down |
| 5(S)-HETE | HMDB0011134 | down | up |
| 9-OxoODE | HMDB0004669 | up | down |
| 9(S)-HODE | HMDB0004670 | up | down |
| 2,3-Dinor-8-iso prostaglandin F1alpha | -- | up | down |
| 5-(2'-Carboxyethyl)-4,6-dihydroxypicolinate | HMDB0006794 | down | up |
| 9(10)-EpOME | HMDB0004701 | down | up |
| 12,13-Epoxy-9-hydroxy-10-octadecenoate | -- | down | up |
| Indoleacetaldehyde | HMDB0001190 | down | up |
| L-Tyrosine | HMDB0000158 | up | down |
